# Promising insights into the health related quality of life for children with severe obesity

**DOI:** 10.1186/1477-7525-11-29

**Published:** 2013-03-01

**Authors:** David T Selewski, David N Collier, Jackie MacHardy, Heather E Gross, Edward M Pickens, Alan W Cooper, Selam Bullock, Marian F Earls, Keeley J Pratt, Kelli Scanlon, Jonathan D McNeill, Kassandra L Messer, Yee Lu, David Thissen, Darren A DeWalt, Debbie S Gipson

**Affiliations:** 1 Division of Nephrology, Department of Pediatrics, University of Michigan, CS Mott Children’s Hospital Room 12-250, 1540 E Hospital Drive, SPC 4297, Ann Arbor, MI 48109-4297, USA; 2 Department of Pediatrics and Pediatric Healthy Weight Research and Treatment Center, Brody School of Medicine, East Carolina University, Greenville, NC, USA; 3 University of North Carolina at Chapel Hill, Chapel Hill, NC, USA; 4 University Pediatrics at Highgate, Durham, NC, USA; 5 Carolina Pediatrics of the Triad, Greensboro, NC, USA; 6 Kids First Pediatrics of Raleigh, Raleigh, NC, USA; 7 Guilford Child Health, Greensboro, NC, USA; 8 Department of Human Services, The Ohio State University, Columbus, OH, USA; 9 Department of Biostatistics, School of Public Health, University of Michigan, Ann Arbor, MI, USA; 10 Department of Psychology, University of North Carolina at Chapel Hill, Chapel Hill, NC, USA; 11 Division of General Internal Medicine, University of North Carolina, Chapel Hill, NC, USA

**Keywords:** Quality of life, Obesity, Patient Reported Outcomes Measurement Information System (PROMIS), Child, Depression

## Abstract

**Background:**

Childhood obesity is a growing health concern known to adversely affect quality of life in children and adolescents. The Patient Reported Outcomes Measurement Information System (PROMIS) pediatric measures were developed to capture child self-reports across a variety of health conditions experienced by children and adolescents. The purpose of this study is to begin the process of validation of the PROMIS pediatric measures in children and adolescents affected by obesity.

**Methods:**

The pediatric PROMIS instruments were administered to 138 children and adolescents in a cross-sectional study of patient reported outcomes in children aged 8–17 years with age-adjusted body mass index (BMI) greater than the 85th percentile in a design to establish known-group validity. The children completed the depressive symptoms, anxiety, anger, peer relationships, pain interference, fatigue, upper extremity, and mobility PROMIS domains utilizing a computer interface. PROMIS domains and individual items were administered in random order and included a total of 95 items. Patient responses were compared between patients with BMI 85 to < 99th percentile versus ≥ 99th percentile.

**Results:**

136 participants were recruited and had all necessary clinical data for analysis. Of the 136 participants, 5% ended the survey early resulting in missing domain scores at the end of survey administration. In multivariate analysis, patients with BMI ≥ 99th percentile had worse scores for depressive symptoms, anger, fatigue, and mobility (*p* < 0.05). Parent-reported exercise was associated with better scores for depressive symptoms, anxiety, and fatigue (*p* < 0.05).

**Conclusions:**

Children and adolescents ranging from overweight to severely obese can complete multiple PROMIS pediatric measures using a computer interface in the outpatient setting. In the 5% with missing domain scores, the missing scores were consistently found in the domains administered last, suggesting the length of the assessment is important. The differences in domain scores found in this study are consistent with previous reports investigating the quality of life in children and adolescents with obesity. We show that the PROMIS instrument represents a feasible and potentially valuable instrument for the future study of the effect of pediatric obesity on quality of life.

## Introduction

Patient-reported outcomes (PRO) related to symptoms, function, and quality of life for children with obesity are an increasingly recognized and integral component of their care. Patient-reported outcomes are now requested as part of treatment summaries submitted to the Food and Drug Administration during trials [1]. Furthermore, valid PROs can contribute to improved patient experiences, satisfaction, interaction of patient and families with physicians, and clinical decision-making [2-4]. To advance the science of PROs in pediatric and adult health, the National Institutes of Health (NIH) funded the Patient Reported Outcomes Measurement Information System (PROMIS; http://www.nihpromis.org). The PROMIS Pediatric multisite initiative created pediatric self-report measures of physical, emotional, and social functioning using modern test theory; the measures offer flexibility of use, including short forms and computer adaptive testing that yield scores on the same metric [5-10].

The PROMIS pediatric measures were developed to capture child and adolescent self-reports across a variety of illnesses experienced by children and adolescents and are currently being tested in longitudinal study designs, including samples of 8 to 17 year olds experiencing cancer, nephrotic syndrome, asthma, sickle cell disease, rheumatoid arthritis, or who are in long-term rehabilitation care (Grant numbers U01AR05218, U01AR057940, and U01AR057929). The PROMIS pediatric measures were designed to be publically available, efficient, precise, and valid across a variety of diseases to assess patient reports of quality of life. PROMIS has been administered to diverse groups of children in clinical and outside of clinical settings to develop a measure that is broadly applicable in a variety of settings [9]. Compared with existing pediatric self-report measures, PROMIS offers more specific measurement of general health domains, but also the flexibility of using various short forms or computerized adaptive testing that all report on the same metric. The intent of the PROMIS pediatric initiative is to advance measurement of health related quality of life and functioning by using the same sensitive measures across chronic illnesses in childhood and adolescence and thus yield new knowledge through direct comparability of scores.

One planned use of the PROMIS pediatric measures is in clinical trials in which measuring change over time is essential to documenting the full impact of treatment on children. A particular benefit of the PROMIS pediatric measures in clinical trials is the strength of standardized measures in repeated administration that accounts for normative developmental changes while maintaining scores on the same metric. Existing research in pediatric obesity has established that children and adolescents are able to validly report on their quality of life, but a variety of measures have been employed [11-15]. Additionally, current measures were primarily developed using classical test methods [16] and not modern test theory or advanced administration and scoring technology. PROMIS pediatric measures were developed using qualitative and quantitative methods (focus groups, expert item review, cognitive interviewing, and item administration to a large sample of children and adolescents) to create banks of items specific to selected domains and quality of life [9,17-20] for use in children 8- to 17- years of age [6,8,10].

Existing literature indicates that children and adolescents with obesity report significantly lower quality of life [11-15,21]. A recent review pooling data from 13 studies has shown obese pediatric patients have significant impairments in physical, social, and emotional functioning [22]. The purpose of this study was to assess the ability of children and adolescents ranging from overweight to severely obese to complete the PROMIS pediatric measures electronically and to establish preliminary estimates of the PROMIS pediatric scores in a pediatric patient sample ranging from overweight to severely obese. We hypothesized that the PROMIS instrument would demonstrate significant impairment in PRO in children with severe obesity compared to overweight and obese children. As a secondary aim, we sought to evaluate differences in PRO by parent-reported child exercise status.

## Methods

### Instruments

The pediatric PROMIS domains in this study included depressive symptoms, anxiety, anger, peer relationships, pain interference, fatigue, upper extremity functioning, and mobility. The definitions of these measures are located at http://www.nihpromis.org/measures/domainframework1. These measures ask participants to respond based on the past 7 days and in terms of a 5-point response scale ranging from ‘never’ to ‘almost always’ in most measures and from ‘with no trouble’ to ‘not able to do’ for physical functioning measures. Higher scores indicate more of the measured domain, which signifies worse functioning for depression, anxiety, anger, fatigue, and pain interference and better functioning for mobility, upper extremity, and peer relationships.

The PROMIS measures were previously tested in a diverse group of children and adolescents with chronic illnesses; characteristics of the measures are available at http://www.assessmentcenter.net or in associated publications [7-9,23]. These assessments confirmed the unidimensionality and the extent to which each item was associated with the measured variable. The measures were found to distinguish levels of the trait for each participant, i.e., high or low anxiety.

Prior publications have shown the PROMIS pediatric measures consistently achieve or exceed a reliability of 0.85 over 2 to 4 standard deviations of the domain under measurement for the short forms [6-8,10]. Using longer item sets than the short forms increases the reliability of measurement.

### Design and administration

This cross sectional study included a convenience sample of 138 children, enrolled at five participating sites, including an academic obesity clinic, three private pediatric practices and a federally qualified health center, between May 2009 and January 2010. The instruments were administered in a computerized web-based format. The total number of PROMIS items completed by all child and adolescent participants was 95. The parents were asked to complete 17 demographic and 29 obesity items. A $10 gift card was provided to the children for participation.

### Sample

The inclusion criteria were age 8–17 years, ability to interact with the computer administered questionnaire, and an age adjusted body mass index (BMI) ≥ 85th percentile. Children and adolescents had to be English-speaking as the PROMIS instrument had not yet been validated in alternative languages. Children and adolescents were excluded if they had any concurrent medical or psychiatric condition that may preclude participation in the study or the presence of a cognitive or other impairment that would interfere with questionnaire completion.

BMI percentiles were calculated using the CDC (Center for Disease Control and Prevention) percentile calculator for children and teens (http://apps.nccd.cdc.gov/dnpabmi/). Percentiles were calculated using age, gender, height, and weight. The participants’ height and weight data were verified using their medical records.

### Personnel training and IRB

Personnel at each site (investigators and study coordinators) received web-based training in study procedures; the study operations manual outlined study conduct, quality control, and recruitment. Ongoing education of site personnel occurred during investigator and coordinator conference calls. Each site had local Institutional Review Board approval. Parental consent and child assent were obtained at study enrollment.

### Study administration

The parent completed the family and medical information form, which included general questions about the caretaker and child demographics as well as disease-specific questions regarding the child. Parents reported whether their child exercised “sometimes or regularly” (greater than or equal to two times per week) or “seldom or never” (less than two times per week). Guardian respondent characteristics included relationship to child and education level.

The children completed the depressive symptoms, anxiety, anger, peer relationships, pain interference, fatigue, upper extremity, and mobility PROMIS scales. In order to reduce response burden, for some domains, the participants completed all items in the banks (depressive symptoms, anxiety, peer relationships, anger) and for others, they were administered only the short form items (upper extremity function, mobility, pain interference, fatigue). Full item banks (e.g., the long forms) were administered for depressive symptoms (15 items), anxiety (20 items), peer relationships (15 items), and anger (11 items) for a total of 61 items. The short forms completed in this study have 8 items, with the exception of fatigue which has 10 items. Each participant received the same combination of short and long forms. The short form items for all domains have been previously published [6]. Using this sampling plan, respondents were administered 95 PROMIS items. Particular care was taken to insure that the children were not assisted by family members or siblings while performing the survey.

Height and weight were measured and recorded by clinical staff during the study encounter.

### Statistical analysis

Descriptive statistics for demographics and child characteristics were calculated. Mean scores were calculated for each of the 8 PROMIS domains by BMI percentile 85th to < 99th (overweight and obese) versus ≥ 99th (severe obesity). The sample was dichotomized into these two BMI groups using a BMI cut point of the 99th percentile both because this divided the sample roughly in half and because the 99th percentile has been proposed as a possible cut-off point for defining severe obesity in childhood [24]. Mean scores were also calculated for each of the 8 domains by parent-reported exercise status. Scores on the PROMIS instruments have been established with a mean of 50 and standard deviation of 10 in the original calibration sample, a mixture of healthy children and those with chronic illnesses [9]. Since the calibration sample was not representative of a specific group (e.g., specific disease, healthy, or general population), the score of 50 does not have specific meaning with respect to the degree of health. In fact, one reason for studies such as this one is to identify the range of scores generated by specifically characterized groups of youth.

Scores were compared between the groups by t-tests. Hierarchical regression analyses were conducted to assess association of child characteristics, recruitment site, parent-reported exercise status, and BMI ≥ 99th percentile with each of the 8 domain scores. For all regression models, independent variables were entered using blockwise selection: demographic items including child’s age, gender, race, and parental education (Block 1); recruitment site (Block 2); and exercise status and BMI ≥ 99th percentile (Block 3, dichotomized as BMI 85th to < 99th percentile and BMI ≥ 99th percentile). Blocks were added in a stepwise fashion, where model 1 included only demographics (Block 1), model 2 included demographics and site (Blocks 1 and 2), and model 3 included demographics, site, exercise status, and BMI percentile (all 3 Blocks). Best model fit was assessed based upon model parameters; results are presented only for best model fit. Missing data were imputed using the expectation maximization (EM) algorithm; Little’s MCAR test was used to check the assumption that the missing data were missing completely at random, an assumption that justifies the use of the EM algorithm [25]. Given the exploratory nature of the study and relatively small sample size, we did not control for the number of statistical tests. All statistical analyses were conducted using Predictive Analytics Software (PASW, formerly SPSS) version 18.0.

## Results

There were 138 participants in this study. The sample by site included 50 children and adolescents from the academic obesity clinic (N = 50), three private pediatric practices (N = 64), and a federally qualified health center (N = 24). The demographics are presented in Table 1. There were 67 (48.9%) patients with BMI ≥ 99th percentile. There were 136 participants included in the analysis; one patient was excluded due to missing BMI data and one patient was excluded due to low BMI (81st percentile).

**Table 1:** Patient demographics for the PROMIS obesity cohort

	**Obesity cohort**	**BMI < 99**	**BMI ≥ 99**	**Comparison of BMI groups**	
**Child demographics**	***N = 138 n (%)***	***N = 70 n (%)***	***N = 67 n (%)***	***t statistic***	***Sig***
**Child’s Gender**					
Female	75 (54.3)	46 (65.7)	29 (43.3)	2.61	0.01
**Child’s Age (yrs)**					
8-12	83 (60.1)	44 (62.9)	37 (55.2)		
13-17	55 (39.9)	25 (35.7)	30 (44.8)		
Age (M, SD)	11.9 (2.7)	11.5 (2.6)	12.4 (2.7)	2.09	0.04
**Child’s Race**					
White	41 (29.7)	29 (41.4)	12 (17.9)	2.98	0.003
Black or African- American	82 (59.4)	28 (40.0)	53 (79.1)	4.95	<0.001
Other	15 (10.8)	13 (18.6)	2 (3.0)	3.06	0.003
**Child’s Ethnicity**					
Hispanic	9 (6.5)	5 (7.1)	4 (6.0)	0.30	NS
**Child’s History of Other Health Problems**					
None	70 (50.7)	34 (48.6)	36 (53.7)		
1 Health Problem	38 (27.5)	24 (34.3)	14 (20.9)		
≥ 2 Health Problem	30 (21.7)	12 (17.2)	17 (25.4)		
Number of Other Health Problems (M, SD)	0.8 (1.0)	0.7 (0.9)	0.9 (1.0)	0.59	NS
Most Common Other Health Problems:*					
Asthma	38 (27.5)	20 (28.6)	17 (25.4)	0.34	NS
ADHD	22 (15.9)	11 (15.7)	10 (14.9)	0.20	NS
Hypertension	13 (9.4)	4 (5.7)	9 (13.4)	1.48	NS
Premature Birth	11 (8.0)	8 (11.4)	3 (4.5)	1.56	NS
Mental Health Disorders	10 (7.2)	3 (4.3)	7 (10.4)	1.33	NS
Diabetes	7 (5.1)	3 (4.3)	4 (6.0)	0.47	NS
**BMI Percentile**					
Healthy Weight (less than the 85th percentile)	1 (0.7)				
Overweight (85th-95th percentile)	11 (8.0)				
Obese					
95th-97th percentile	30 (21.9)				
98th percentile	28 (20.4)				
99th percentile	49 (35.8)				
> 99th percentile	18 (13.1)				
Missing	1 (0.7)				
	**Obesity Cohort**	**BMI < 99**	**BMI ≥ 99**	**Comparison of BMI groups**	
**Guardian's Relationship to the Child**					
Parent	129 (93.5)	66 (94.3)	62 (92.6)	0.77	NS
Grandparent	4 (2.9)	2 (2.8)	2 (3.0)	0.03	NS
Guardian or Other	5 (3.6)	2 (2.9)	3 (4.5)	1.03	NS
**Guardian Education Level**					
≤ 8th grade or Some High School	11 (8.0)	3 (4.3)	8 (12.0)	1.62	NS
High School Degree/GED	23 (16.7)	11 (15.7)	12 (17.9)	0.30	NS
Some College/ Technical Degree	65 (47.1)	29 (41.4)	35 (52.2)	1.36	NS
College or Advanced Degree	39 (28.2)	27 (38.5)	12 (17.9)	2.80	0.006
**Recruitment Site**					
Academic Obesity Center	50 (36.2)	14 (20.0)	36 (53.7)	4.27	<.001
Private Pediatric Practice	64 (46.4)	43 (61.4)	21 (31.3)	3.59	<.001
Federally Qualified Health Center	24 (17.4)	13 (18.6)	10 (14.9)	0.61	NS
**Child Exercise Status**					
Regularly (5–7 times per week)	37(26.8)	18 (25.7)	19 (28.4)	0.30	NS
Sometimes (2–4 times per week)	63(45.7)	30 (42.9)	33 (49.3)	0.67	NS
Seldom (0–1 time per week)	37(26.8)	22 (31.4)	14 (20.9)	1.27	NS
Missing	1(0.7)	0	1 (1.5)		

* Parents reported more than 1 condition for some children; there were many other conditions reported in lower frequency (< 3%) than the conditions listed.

*NS*: Not Significant (*p* < 0.10).

### Feasibility

The average time (calculated by the difference between the time stamp on the first and last items) for those who completed the survey was 32.4 minutes. Of the 136 participants, seven participants (5%) had missing scores for at least one of the PROMIS measures. Six of these seven participants ended the survey early, so that measures administered at the beginning of the survey had scores, but measures administered at the end of the survey were missing responses. Because the PROMIS measures were administered in random order, missing scores were spread across the domains. For the remaining participant, an entire scale was skipped in the middle of the survey (pain interference). A missing value analysis examined patterns of missingness and did not reject the hypothesis that the data were missing completely at random (Little’s MCAR test, *Χ*^*2*^(34) = 39.83, *p* = 0.28), when the other variables (e.g., PROMIS pediatric measure, child gender, age, recruitment site, exercise status, and BMI) were considered. There was no difference in age between the group with no missing PROMIS scores and the group with one or more missing scores (t = 1.07, *p* = 0.29).

### Descriptive findings

In analyses comparing the PROMIS domain scores for participants with BMI 85th to 99th percentile with those ≥ 99th percentile, the domain scores for anger (47.0 vs. 50.7, *p* = 0.04), fatigue (43.2 vs. 47.6, *p* = 0.02) and mobility (52.3 vs. 48.4, *p* = 0.001) were significantly different. There were no significant differences found for the domains of anxiety, peer relationships, and upper extremity (Table 2).

**Table 2:** Analyses of the PROMIS instrument by patient obesity

**Domain**	**Age related BMI percentile**	**N**	**Mean**	**Std. Dev**	**t**	***p *value**
Depressive Symptoms	< 99	69	46.4	8.4	−1.8	0.08
	≥ 99	67	49.2	9.9		
Anxiety	< 99	69	46.6	9.8	−0.9	0.36
	≥ 99	67	48.3	11.7		
Anger	< 99	69	47.1	9.9	−2.0	0.04
	≥ 99	67	50.7	10.4		
Peer Relationships	< 99	69	48.1	8.6	1.3	0.20
	≥ 99	67	46.1	9.0		
Pain Interference	< 99	69	46.4	9.4	−1.7	0.10
	≥ 99	67	49.1	9.2		
Fatigue	< 99	69	43.2	9.6	−2.4	0.02
	≥ 99	67	47.6	11.6		
Upper Extremity	< 99	69	51.4	7.1	0.3	0.75
	≥ 99	67	51.0	7.3		
Mobility	< 99	69	52.3	6.2	3.2	0.001
	≥ 99	67	48.4	7.6		

*BMI*: Body Mass Index.

Further analyses found that the PROMIS scores were significantly better in the domains of depressive symptoms (46.9 vs. 50.4, *p* = 0.05), anxiety (46.4 vs. 50.5, *p* = 0.05), and fatigue (44.0 vs. 49.7, *p* = 0.007) for those whose parents reported the child exercised sometimes or regularly compared with those whose parent-reported exercise was seldom or never (Table 3). There was no association between BMI (≥ 99th percentile vs. 85th to 99th percentile) and exercise status (t = 1.02, *p* = 0.31).

**Table 3:** Analyses of the effect of exercise status using the PROMIS instrument

**Domain**	Child exercise recorded*	**N**	**Mean**	**Std. Dev**	**t**	***p *value**
Depressive Symptoms	Seldom or never	35	50.4	9.4	2.0	0.05
	Sometimes or regularly	100	46.9	9.1		
Anxiety	Seldom or never	35	50.5	12.0	2.0	0.05
	Sometimes or regularly	100	46.4	10.2		
Anger	Seldom or never	35	51.7	10.9	1.9	0.06
	Sometimes or regularly	100	47.9	10.0		
Peer Relationships	Seldom or never	35	46.8	9.1	−0.3	0.78
	Sometimes or regularly	100	47.2	8.8		
Pain Interference	Seldom or never	35	49.8	9.5	1.5	0.15
	Sometimes or regularly	100	47.1	9.3		
Fatigue	Seldom or never	35	49.7	11.4	2.8	0.007
	Sometimes or regularly	100	44.0	10.2		
Upper Extremity	Seldom or never	35	51.1	7.6	−0.5	0.96
	Sometimes or regularly	100	51.1	7.1		
Mobility	Seldom or never	35	50.2	8.1	−0.1	0.89
	Sometimes or regularly	100	50.4	6.9		

* Exercise coded as “seldom or never” group reported participation in physical activity < 2 times per week.

The academic obesity clinic had a higher number of children and adolescents with BMI ≥ 99th percentile while the private pediatric practices had a higher number of children and adolescents with BMI 85th to 99th percentile, (χ^2^(2, *N* = 136) = 17.05, *p* < 0.001).

As hypothesized, mean scores on the PROMIS pediatric measures differed significantly between the participants with BMI 85th to 99th percentile compared to those with BMI ≥ 99th percentile in several of the PROMIS domains (Table 4), even after adjusting for the effects of demographic variables and exercise status.

**Table 4:** Regression coefficients and confidence intervals for predictors of the PROMIS scores

	**Domains**				
	**Depressive symptoms**	**Anxiety**	**Anger**	**Fatigue**	**Mobility**
	**β [95% Cl]**	**β [95% Cl]**	**β [95% Cl]**	**β [95% Cl]**	**β [95% Cl]**
Age related BMI ≥ 99th Percentile	4.3 [0.6,8.0]*	1.1 [−3.1,5.3]	5.2 [1.1, 9.3]*	4.3 [0.1,8.5]*	−4.9 [−7.8,-2.0]**
Child Exercise	−4.3 [−7.9, −0.7]*	−5.6 [−9.7,−1.4]**	−3.9 [−7.9,0.2]	−7.5 [−11.7,-3.4]***	0.8 [−2.0, 3.6]
Age	−0.3 [−1.0,0.3]	−0.8 [−1.5, −0.1]*	0.4 [−0.3, 1.1]	−0.6 [−1.3, 0.1]	0.2 [−0.3, 0.7]
Gender	−5.1 [−8.3, -2.0]**	−4.1 [−7.7, −0.5]*	−4.8 [−8.4,-1.3]**	−0.1 [−3.7, 3.5]	2.6 [0.2, 5.1]*
Black	−0.7 [−4.5, 3.0]	2.2 [−2.1, 6.5]	0.9 [−3.3, 5.1]	2.7 [−1.6, 6.9]	2.2 [−0.7, 5.1]
Race Other	0.3 [−5.0, 5.7]	−2.3 [−8.5, 3.8]	−1.0 [−7.1, 4.9]	−1.4 [−7.6, 4.7]	0.4 [−3.8, 4.6]
Parent Education	−2.9 [−6.5, 0.8]	−5.2 [−9.4, -1.0]*	0.0 [−4.1, 4.1]	−5.2 [−9.4, -1.0]*	1.2 [−1.6, 4.1]
Recruitment Site:					
Academic Obesity Center	1.5 [−2.2, 5.3]	3.8 [−0.5, 8.0]	−3.4 [−7.6,0.7]	−0.3 [−4.5, 4.0]	−2.0 [−4.9, 0.9]
Federally Qualified Health Center	1.3 [−3.3, 5.8]	1.3 [−3.9, 6.5]	0.3 [−4.8, 5.3]	−1.0 [−6.2, 4.2]	−1.8 [−5.3, 1.8]

- Child Exercise coded as “sometimes or regularly” group reported participation in physical activity ≥ 2 times per week.

- Gender coded as male.

- Parent Education coded as “some college or more”.

Expressed as 95% Confidence Intervals.

* *p* < 0.05.

** *p* < 0.01.

*** *p* < 0.001.

Hierarchical regression analyses showed that the models that included the demographics, site, BMI percentile ≥ 99th percentile, and parent-reported exercise status variables yielded the best fits for the PROMIS domains of depressive symptoms (F = 2.54, *p* = 0.01), anxiety (F = 2.96, *p* = 0.003), anger (F = 2.46, *p* = 0.013), fatigue (F = 3.07, *p* = 0.002), and mobility (F = 2.37, *p* = 0.02). Children with BMI ≥ 99th percentile on average had about a 4 to 5 point worse score for depressive symptoms, anger, fatigue, and mobility (*p* < 0.05, Table 4). Children who exercised sometimes or regularly reported fewer depressive symptoms by 4 points, anxiety by 5.6 points, and fatigue by 7.5 points. Males on average had lower scores for depressive symptoms, anxiety, and anger and higher scores for mobility (*p* < 0.05). Race and recruitment site did not significantly influence scores in any domain. These models were not significant for the domains of upper extremity, peer relationships, or pain interference.

We examined the possibility that gender moderated the effect of obesity on PROMIS outcomes by adding a gender-obesity interaction term to the regression models. We did not find a significant interaction effect for depressive symptoms, anxiety, anger, or fatigue. However, a significant interaction between gender and obesity was identified in the mobility domain (β = 6.28 [1.52, 11.03], *p* = 0.01). For males, the relationship between obesity and mobility was not significant. For females, there was a strong relationship between obesity and mobility (*p* < 0.001), with females in the severe obesity group (99th percentile or higher) showing significantly lower levels of mobility.

## Discussion

Pediatric obesity is a major public health concern that will serve as a challenge for decades to come. The psychosocial implications of severe obesity on children and adolescents have been increasingly recognized and studied but not routinely addressed in clinical practice. Our study provides the initial evaluation of the feasibility and utility of the PROMIS pediatric measures in children and adolescents with obesity. We show that PROMIS pediatric scales are sensitive to children with severe obesity when compared with overweight children, consistent with previous publications [11-13]. These findings also indicate the feasibility of administering these measures to children and adolescents during clinical encounters. The PROMIS instruments provide a potentially valuable tool to researchers and clinicians who seek to study the psychosocial and physical functioning implications of obesity and patient responses to therapy.

Findings from this study indicate that 95% of children and adolescents who are overweight to severely obese are able to complete the PROMIS pediatric measures during clinic visits using personal computers. Further, the PROMIS pediatric measures were completed with low rates of missingness, and the missingness appeared to be random with regard to disease and respondent demographic characteristics. Our rates of missingness are slightly higher or similar to rates in studies where children were interviewed face-to-face or during telephone calls regarding symptoms and quality of life (0.2 to 2.8%) [1,26-28]. Our findings also suggest that the length and number of items is an important factor in successful completion of the instrument as the domains that were not completed occurred at the end of the questionnaire.

Findings from our study are consistent with previous reports about worse quality of life for children with BMI ≥ 99th percentile. In 2003, Schwimmer and colleagues demonstrated that severely obese patients at an academic pediatric obesity clinic had significant impairments in physical, psychological, emotional, social, and school functioning as assessed by the PedsQL ^TM^ 4.0 [12]. In 2005, Williams and colleagues reported a larger community-based cross-sectional study that found statistically significant but smaller relationships between patient weight and quality of life utilizing the PedsQL ^TM^ 4.0 [11]. Our findings are consistent with these studies using the PROMIS instruments, including poorer functioning in the domains of depressive symptoms, anger, fatigue, and mobility for children and adolescents with BMI ≥ 99th percentile. The findings of increased burden in these domains parallels those measured by the composite psychosocial and physical components of the PedsQL ^TM^ 4.0 utilized in the previously mentioned studies [11,12]. Our study differs from previous publications because the comparative samples range from overweight to obese (BMI 85th to <99th percentile). The PROMIS instruments detected quality of life differences in these groups consistent with those previously published utilizing a variety of quality of life measures. Our findings show that the PROMIS pediatric measures can be used in children with obesity to study quality of life.

In adults and children with varying degrees of obesity, it is clear that those with severe obesity are at higher risk for symptoms associated with depression compared with those not obese [29-31]. The data in community-based samples regarding depressive symptoms in obese pediatric patients are conflicting. In 2005, Sjoberg and colleagues reported an association of depressive symptoms and clinical depression with severe obesity in a population-based survey [32]. Among severely obese youth seeking treatment at an academic pediatric obesity center, both youth and caregiver depression was found to be predictive of having an “at risk” general quality of life score [33]. In our study, depression scores were worse in severely obese children and adolescents relative to those who were overweight to obese. The inclusion of participants from an academic obesity clinic, private pediatric practices, and a federally qualified health center suggests that this finding is generalizable to other severely obese children and adolescents.

The importance of increasing physical activity for overweight and obese children to lower weight and improve cardiovascular health is accepted. Recent publications have shown improved neurocognitive function in previously sedentary overweight patients who participate in regular physical exercise. One study found a specific improvement in executive function and brain activation in overweight patients exercising 20–40 minutes per day [34]. The benefits of exercise and activity on self-image in obese pediatric patients have been demonstrated in small single center studies [35]. As a secondary component to the validation of the PROMIS instrument in children with obesity, we sought to evaluate the relationship between parent reported physical activity and PROs. We demonstrated that children who exercised as little as two times per week had better scores on the PROMIS domains for depressive symptoms, anxiety, and fatigue. While a causal relationship cannot be established from this observation, exercise may improve depressive symptoms and quality of life for obese children and adolescents in addition to and potentially independent from weight loss and cardiovascular health. Our findings suggest that changes in the depressive symptoms, anger, anxiety and fatigue domains over time may be valuable outcome measures for interventional clinical trials related to obesity.

An unexpected finding in our study was that children with a BMI ≥ 99th percentile reported lower overall mobility. In the multivariate analysis, BMI ≥ 99th percentile was the most significant predictor of PROMIS mobility scores. There were eight questions that comprised the mobility short form items including, “I could get up from the floor” and “I could stand up by myself” [6]. The implications of such findings are profound when one considers the potential effect diminished mobility has for obese patients as physicians attempt to encourage exercise as treatment. This is particularly alarming when one takes into account the fact that our comparative patient sample ranged from overweight to obese (BMI 85th to <99th percentile). A potential explanation of our findings is found in recent studies demonstrating that obese adolescents have significantly altered biomechanics in all joints in the lower extremity [36,37]. Although these findings were modified by an interaction between gender and obesity, they warrant further study and highlight a potential importance of early intervention. Taken together these findings reinforce the potential importance of developing aggressive interventions prior to the development of severe obesity and suggest that the PROMIS instrument is sensitive to patient reported mobility challenges in obese children and adolescents.

Some limitations of this study should be noted. The first is that the comparative patient sample did not include healthy individuals, and the instrument was scored based on the calibration sample, which included healthy and chronically ill children. The comparative patient sample in this study was composed of participants who qualify as overweight to obese. This shows the strength of the PROMIS instrument in that it was sensitive enough to detect clinically meaningful differences across samples with relatively small differences in BMI. We acknowledge that utilizing a BMI cutoff of ≥ 99th percentile has some shortcomings and instability [38], but at this time, this cut-off is the most accepted and utilized measure in the field. Another limitation is that due to the exploratory nature of the analyses and relatively small sample size, we did not control for the number of tests in our statistical analyses.

The PROMIS instrument is limited by the age of participants who can provide self-report. The PROMIS measures were designed to collect patient-reported outcomes directly from children ages 8 and above. Only English language versions of the pediatric PROMIS instrument were available at the time of this study. The availability and validity of other language versions will be important for future broad scale validity of PROMIS. A longitudinal study validating the responsiveness of the PROMIS instrument domains over time or in response to changes in BMI should be performed to further validate the instrument.

Specific strengths of this study include the inclusion of children and adolescents from a wide variety of socio-economic and healthcare environments by including representation from academic referral, private practice, and community-based health clinics. This represents one of the broadest samplings studying quality of life in childhood obesity.

## Conclusions

Electronic administration of PROMIS pediatric measures is feasible, and this study begins to establish instrument utility in overweight and obese children and adolescents. We show that successful administration of the PROMIS instrument in overweight to severely obese children and adolescents in an outpatient setting is feasible with minimal missing data. The PROMIS instrument demonstrated a higher burden of depressive symptoms, anger, fatigue, and mobility for children and adolescents with BMI ≥ 99th percentile compared to those with BMI in the 85 to < 99th percentiles, consistent with previous literature on quality of life. In addition we demonstrated a relationship between physical activity and patient perceptions of depressive symptoms, anxiety, and fatigue, adjusting for the contribution of BMI. We showed that the PROMIS instrument represents a potentially valuable instrument for the future study of the effect of interventions in pediatric obesity.

## Abbreviations

PRO: Patient reported outcomes; PROMIS: Patient Reported Outcomes Measurement Information System; BMI: Body Mass Index

## Competing interest

The authors declare that they have no competing interests. The authors have no financial relationships or conflicts of interest relevant to this article to disclose.

## Authors’ contributions

DTS, KLM, YL: Drafting the article or revising it critically for important intellectual content. JMacH, KS, JMcN, EMP, AC, SB, MFE: Substantial contributions to conception and design, acquisition of data, or analysis and interpretation of data. HEG: Substantial contributions to conception and design, acquisition of data, or analysis and interpretation of data. Drafting the article or revising it critically for important intellectual content. DNC, KP, DT, DADeW and DSG: Substantial contributions to conception and design, acquisition of data, or analysis and interpretation of data. Drafting the article or revising it critically for important intellectual content; and final approval of the version to be published. All authors read and approved the final manuscript.
